# The protease-activated receptors are expressed in glioblastoma and differentially modulate adherent versus stem-like growth of LN-18 GBM cells

**DOI:** 10.3389/fonc.2025.1582996

**Published:** 2025-07-22

**Authors:** Sandra Bien-Möller, Antonia Grober, Judith Albrecht, Heiko Paland, Kerstin Weitmann, Angela Bialke, Sascha Marx, Silke Vogelgesang, Mladen V. Tzvetkov, Wolfgang Hoffmann, Henry W. S. Schroeder, Bernhard H. Rauch

**Affiliations:** ^1^ Department of Pharmacology, University Medicine Greifswald, Greifswald, Germany; ^2^ Clinic of Neurosurgery, University Medicine Greifswald, Greifswald, Germany; ^3^ Department of Community Medicine, University Medicine Greifswald, Greifswald, Germany; ^4^ Department of Neuropathology, University Medicine Greifswald, Greifswald, Germany; ^5^ Pharmacology and Toxicology, University Medicine Oldenburg, Carl von Ossietzky University Oldenburg, Oldenburg, Germany

**Keywords:** glioblastoma, protease activated receptors, PARs, neurospheres, stem-like cells

## Abstract

**Background:**

Glioblastoma (GBM) remains the most aggressive and common malignant brain tumor in adults, often accompanied by venous thromboembolism due to hypercoagulability. Protease-activated receptors (PAR1-4) are thought to influence GBM progression, which in this study led to examine their expression in both tissue from GBM patients and in a GBM cell model.

**Methods:**

Using quantitative PCR and immunoblot analyses, we investigated the expression of PAR1-4 in human GBM samples compared to non-malignant brain and evaluated its role in patient survival. In addition, the expression of PAR1-4 in adherent LN-18 GBM cells in comparison to their stem cell-like neurosphere counterparts was analyzed. Finally, the influence of PAR1-4 modulation by specific agonists and antagonists on cell viability was investigated using this GBM cell model.

**Results:**

PAR1-4 mRNA levels were significantly higher in GBM than in non-tumoral brain tissue, though this did not affect patient survival. Notably, PAR4 protein levels were lower in GBM, while PAR1, 2, and 3 were unchanged. However, high PAR1 protein levels were linked to poorer patient survival, with a similar trend observed for PAR4, though not statistically significant. Patients with high levels of both PAR1 and PAR3 or PAR4 faced an even greater risk of poor outcomes, but the most severe prognosis was seen in those patients with high PAR3 and PAR4 protein level. In stem-like LN-18 GBM neurospheres, PAR1-4 mRNA was significantly increased, with PAR3 protein elevated and PAR4 reduced. Inhibition of PAR1, PAR2, or PAR4 reduced the viability of adherent GBM cells but not stem-like neurospheres.

**Conclusion:**

These findings suggest that PARs impact GBM patient survival and that tumor stem cells may respond differently to PAR inhibition compared to conventional tumor cells.

## Graphical Abstract

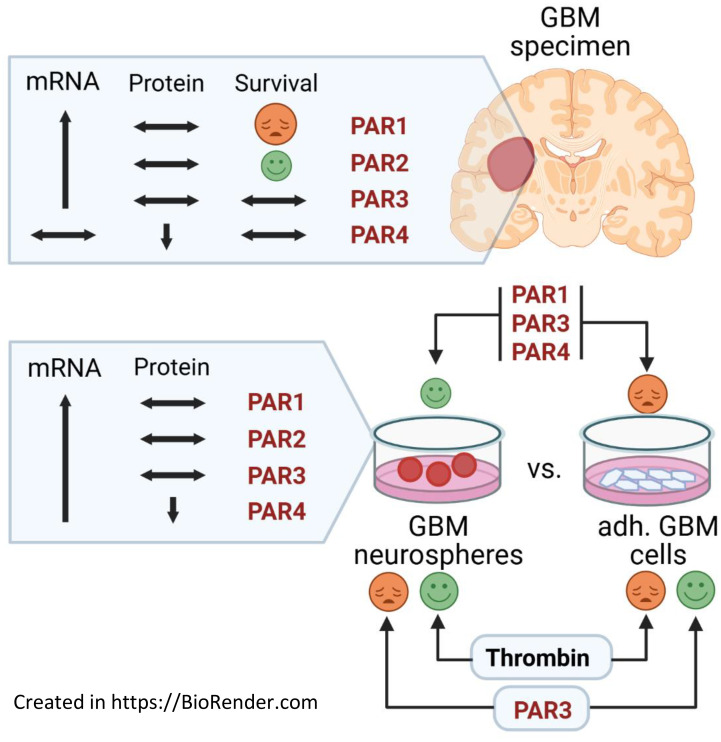

## Introduction

Glioblastoma (GBM) is the most common and aggressive primary brain tumor in adults, and is fatal in almost all patients. Despite a multimodal therapy based on a combination of maximal surgical resection, radiation and chemotherapy with temozolomide, the median overall survival is only about 15 months with a 5-year survival rate of 6.9% (1, 2). Thus, identification and evaluation of new targets to improve the current therapeutic regime and the overall survival of the patients is urgently needed.

Besides the extremely rapid and infiltrative growth behavior, neovascularization and venous thromboembolism are characteristic for GBM and also lead to tumor-related intracranial hemorrhage and subsequently higher thrombin burden in GBM patients (3). Previous studies have linked thrombin to tumor cell adhesion to platelets, endothelial cells, and subendothelial matrix proteins, leading to migration and spontaneous metastasis (4, 5). It has been shown that the central nervous system (CNS) is the only site where thrombin is expressed outside the liver, being involved in brain development, protection and regeneration (4). The serine protease thrombin exerts its cellular function, which include cell cycle progression, cell growth, migration, and proliferation, via G-protein-coupled receptors called protease-activated receptors (PARs). The PAR receptor family consists of 4 members (PAR1-4) that are activated by a biphasic cleavage process of the extracellular N-terminus (6). Initially, thrombin was thought to interact with PAR1, PAR3, and PAR4, whereas PAR2 is activated by other serine proteases, including trypsin and mast cell tryptase (7). But recently, evidence shows that thrombin, at sufficient concentrations to be achieved at sites of brain injury or in the tumor environment, is also capable of activating PAR2 and triggering its downstream signaling pathways (8). PAR signaling particularly occurs via activation of phospholipase C, formation of inositol triphosphate and diacylglycerol, subsequent release of Ca2+, and activation of protein kinase C, but also by activation of phosphatidylinositol 3-kinase and mitogen-activated protein kinase (9).

PARs are expressed on a variety of physiological and degenerative tissues, and PARs are found in the vasculature and throughout the CNS, especially in neurons, microglia, astrocytes, and oligodendrocytes (10). It has been demonstrated that PAR expression correlates with cancer malignancy, and clinical studies show that anticoagulant treatment is beneficial in cancer patients (11).

Gliomas also express functionally active PAR1 and PAR2, which has been demonstrated on both primary and commercially available cell lines (12–14). Thrombin is known to act as a growth factor and triggers proliferation via PAR1 (7). Numerous findings support the involvement of the thrombin-PAR1 pathway in glioma pathology (15). Increased expression of PAR1 within tumor cells and within tumor blood vessels depending upon the tumor area was found in GBM samples suggesting a functional role of PAR1 in GBM cell malignancy and angiogenesis (16). Immunohistochemical analysis of malignant gliomas showed an enhanced PAR1 expression with increasing WHO grade (17). Overexpression of PAR1, the primary thrombin receptor in the CNS, correlates with larger tumor masses and tumor-induced brain edema in an *in vivo* rat glioma model (18). Conversely, knockdown of PAR1 inhibited tumor growth *in vitro* and resulted in prolonged survival in a mouse model (19). Dabigatran, a selective direct thrombin inhibitor, antagonizes growth, cell-cycle progression, migration, and endothelial tube formation induced by thrombin in breast and glioblastoma cells (20).

Regarding PAR2, this receptor could also play an important role in GBM via the modulation of VEGF production by the MAPK/ERK1/2 pathway (14). Activation of PAR2 reduces glioblastoma cell apoptosis (21), and inhibition of tissue factor/PAR2 signaling limits proliferation, migration and invasion of malignant glioma cells (22). Only a few and conflicting results are available on the expression of PAR3 and PAR4 in malignant gliomas. Ostrowska and colleagues detected a functionally active PAR3 on glioma cells (23). Others could not detect PAR3 and PAR4 in glioma cells at either the protein or mRNA level (12, 24).

Currently, there is no published study that comprehensively investigates the expression of PARs in human GBM tissue in terms of their impact on patients´ survival. Therefore, we analyzed the expression of PAR1–4 in human GBM samples in comparison to non-malignant brain and evaluated their role for patient´s survival. Furthermore, expression of PAR1–4 was investigated in adherent LN-18 glioblastoma cells and their stem cell-like neurosphere counterparts.

## Results

### mRNA expression of PAR1, PAR2 and PAR3 is elevated in GBM mRNA but is not associated with patient´s survival time

**Figure 1:**
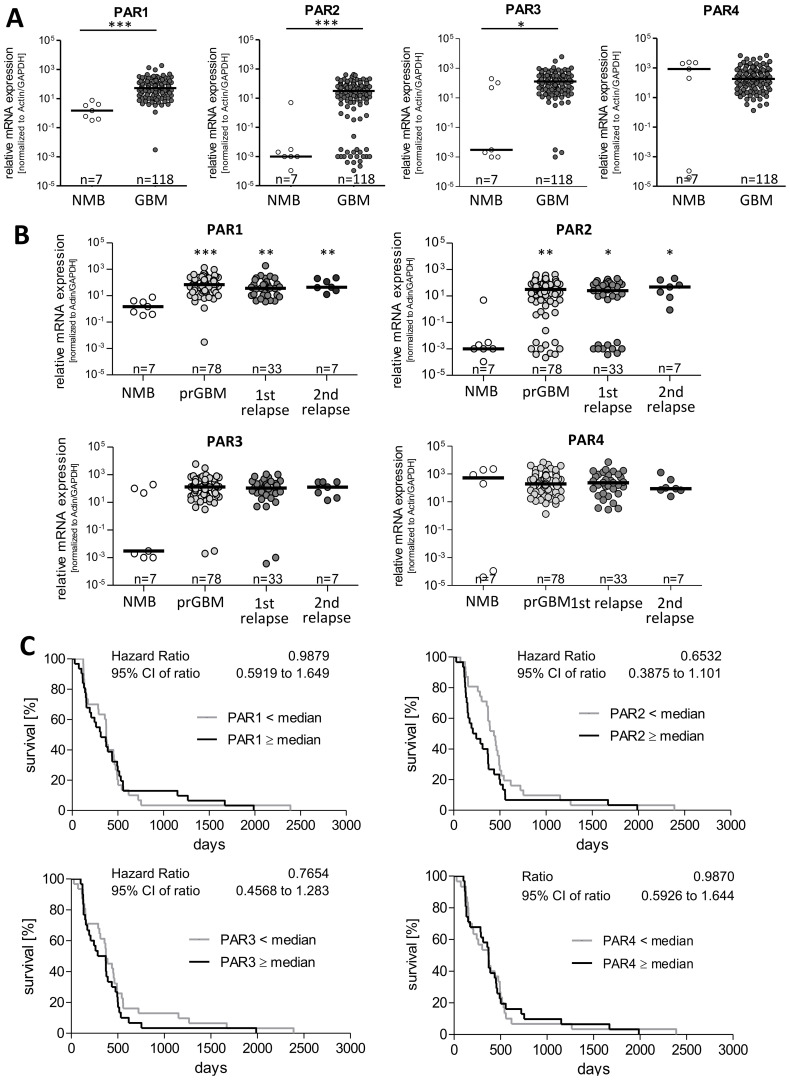
PAR1–4 mRNA expression in GBM specimen and its association with patients´ overall survival time. **(A)** Comparison of PAR1, PAR2, PAR3 and PAR4 mRNA expression in non-malignant brain (NMB, n=7) and all analyzed GBM samples (both primary and relapsed GBM, n=118). **(B)** Subdivision of GBM specimen in primary GBM (prGBM, n=78), first (1^st^, n=33) and second (2^nd^, n=7) relapses and comparison with NMB. (A+B) Gene expression was measured by qPCR. Each mRNA level of the target genes (PAR1-4) was normalized to the mean of GAPDH and β-actin using the 2^-ΔΔct^ method. Data are shown as scatter plots representing the median as horizontal bars. Mann Whitney U test, *p < 0.05 and ***p < 0.001 for **(A)** and OneWay ANOVA/Kruskal Wallis test with Dunn’s Multiple Comparison Test, *p < 0.05, **p < 0.01 and *** p < 0.001 for **(B)**. **(C)** Kaplan Meier survival analyses of PAR1–4 mRNA expression in GBM patients. Association of the relative mRNA expression of each single PAR receptor with the survival time of patients with primary GBM. The patients were divided into two subgroups depending on the median gene expression. No significant association was found.

To elucidate the impact of PARs on GBM pathogenesis, we analyzed mRNA expression of all four PAR subtypes in GBM specimens (n=118) and seven non-malignant brain tissues (NMB). Results are shown in **Figure 1A** and are expressed in the following text as median and 25-75% percentiles. The expression of PARs was not always detectable in the non-tumoral brain tissue even if the housekeeping genes were normally expressed. PAR expression was set to 0.001 in these samples and included in the analyses, which explains the wide variation in non-tumoral brain samples. PAR1 mRNA was significantly elevated from a median value of 1.5 [0.39-4.03] in non-tumoral brain tissue to 52.8 [19.87-161.7] in GBM. A similar increase was observed for PAR2 mRNA with a median of 0.001 [0.001-0.003] in non-tumoral brain tissue and of 31.01 [4.03-78.72] in GBM, respectively. PAR3 mRNA was also strongly up-regulated from 0.003 [0.001-103.7] in non-malignant brain to 127.7 [35.2-325.1] in GBM, respectively. In contrast, PAR4 mRNA showed a trend to be reduced in GBM specimen with a median value of 180.4 [51.4-596.7] compared to a median of 842.3 [0.0001-2263] in non-tumoral brain specimen, but this failed to get statistically significant. Of note, subdividing GBM samples into primary and relapsed tumors (1^st^ and 2^nd^ relapse) revealed an elevated expression of PAR1, PAR2 and PAR3 without any significant differences between these tumor specimens (**Figure 1B**).

Furthermore, there was no correlation between mRNA expression of PARs and the age at diagnosis (**Supplementary Figure S1A**). To analyze whether sex specific variation in PAR expression exist, patient´s samples were subdivided in female and male ones showing no significant differences between the gender (**Supplementary Figure S1B**).

To examine whether a correlation between the expression of the single PAR subtypes is present in GBM specimen, we performed Spearman’s correlation analyses. As demonstrated in **Supplementary Figure S1C**, only a moderate positive correlation was found between the mRNA level of PAR1 and PAR2 as well as slight negative correlation between PAR1 and PAR4 mRNA expression. For all other combinations, a significant correlation could not be found.

To investigate a potential impact of PAR mRNA expression on patient´s prognosis, we performed Kaplan Meier survival analyses. For this, patients were subdivided in to subgroups depending on the respective PAR subtype: < median versus ≥ median PAR mRNA expression.

As shown in **Figure 1C**, the survival curves did not show any association between mRNA expression of the individual PAR subtype and the survival of GBM patients. We also investigated whether the survival of GBM patients was affected when the mRNA levels of two different PAR subtypes showed either high or low expression at the same time. This combined analyses also revealed no significant association with the patients´ survival and therefore do not indicate direct additive effects.

### Expression of PAR1–4 at the protein level and their impact on survival of GBM patients

**Figure 2:**
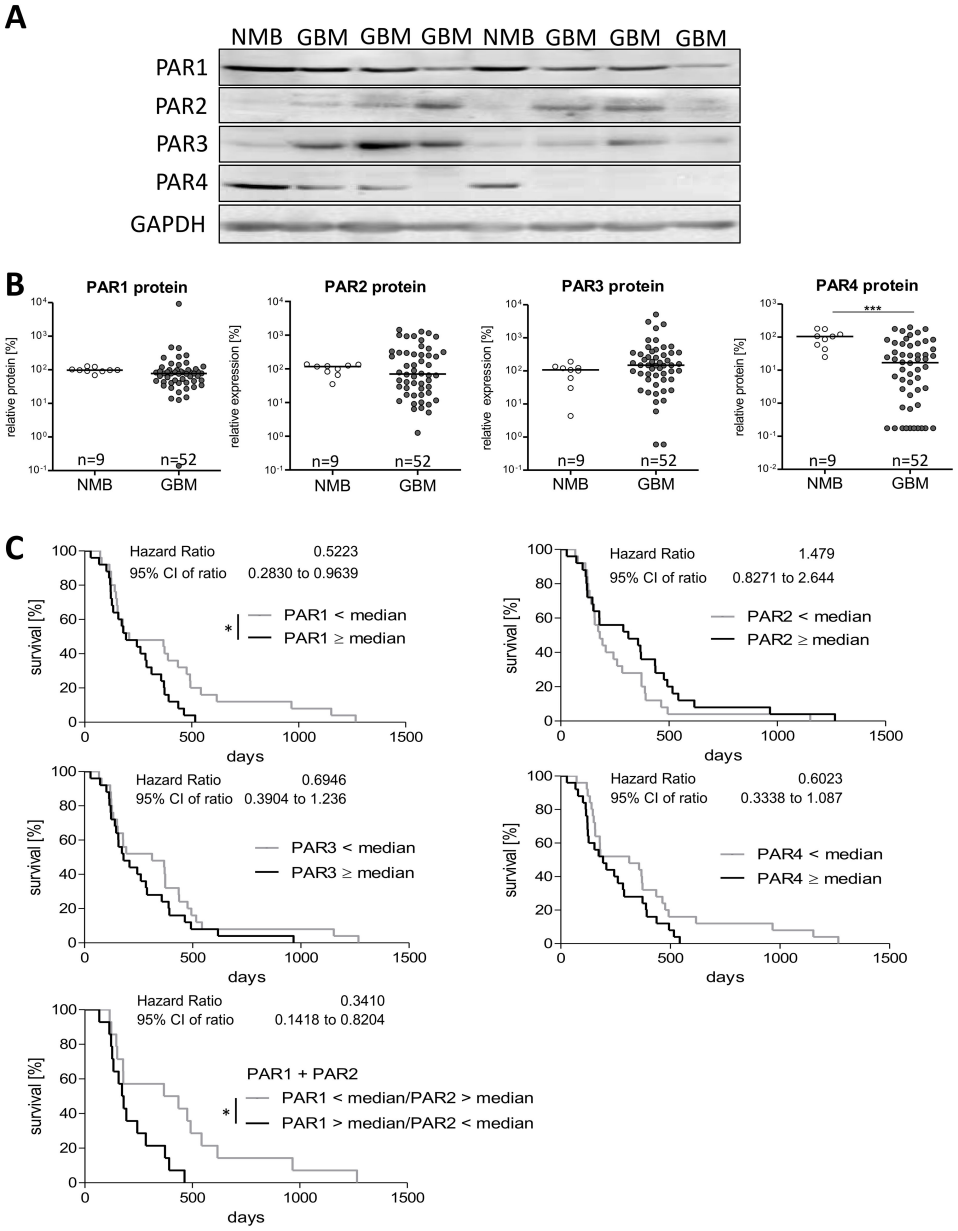
Protein expression of PAR1–4 in GBM patients´ samples. **(A)** Representative immunoblots of PAR1–4 in GBM tissue in comparison to non-malignant brain samples (NMB). Detection of GAPDH was used as loading control for normalization of the respective PAR protein level to a housekeeping protein. **(B)** Relative protein expression of PAR1–4 in GBM and NMB after densitometric evaluation and normalization to GAPDH. Data are shown as scatter plots representing the median as horizontal bars. Mann Whitney U test, ***p < 0.001. **(C)** Kaplan Meier survival analyses of PAR1–4 protein expression in GBM patients. Association of the relative protein expression of each single PAR receptor with the survival time of patients with primary GBM. The patients were divided into two subgroups depending on the median protein expression. The protein content of the respective PARs was determined by western blotting and normalized to the housekeeping protein GAPDH. Log-rank (Mantel-Cox) Test, *p < 0.05.

By using immunoblot analysis, we investigated the protein expression of PAR1–4 in 52 GBM specimens and nine non-malignant brain tissues. In **Figure 2A**, representative blots of each PAR subtype in two NMB samples and six GBM specimen are presented, the respective statistical analyses are seen in **Figure 2B**. The majority of NMB and GBM samples showed a high expression of PAR1 protein without any significant differences between non-tumoral brain specimen [91.68, 75.04-123.7] and GBM [77.58, 42.03-98.90]. Protein expression of PAR2 and PAR3 was rather low in NMB, and PAR2 as well as PAR3 protein showed a large inter-individual variability among GBM patients. For both PAR2 and PAR3 protein, also no significant difference was found between NMB and GBM samples [PAR2: 95.52, 68.4-125.2 versus 70.27, 21.15-301.1; PAR3: 92.18, 51.14-146.4 versus 148.3, 55.04-351.3]. In contrast, across all NMB samples, we noted a high median level of PAR4 protein [98.15, 51.3-128.3] which was significantly decreased in GBM specimens to 16.9 [1.10-60.34].

Correlation analyses for mRNA and protein levels of PARs (**Supplementary Figure S2B**) revealed no significant association for all PAR subtypes, but a trend to a negative correlation was found for PAR1 mRNA and protein (r^2^ -0.264, p=0.088). To determine if PAR protein level is influenced by the age at diagnosis, we again performed a Spearman correlation analysis. As shown in **Supplementary Figure S2C**, the correlation data did not suggest any impact of patient´s age on PAR protein expression. Also, no significant difference in PAR protein expression was found between female and male GBM samples (**Supplementary Figure S2D**).

As correlation analyses between mRNA and protein content of PARs showed no significant association, there might be different effects of the PAR protein levels on patients´ prognosis. Thus, we also investigated the influence of PAR subtype protein content on patients´ survival time using Kaplan Meier survival analyses. Again, we divided the patient cohort depending on the median protein expression in to two subgroups: < median versus ≥ median PAR protein expression. The results are shown in **Figure 2C**. Despite no significant impact of PAR mRNA expression on survival time of GBM patients, we observed a significant association between a high PAR1 protein level and a worse overall survival time (Hazard Ratio: 0.522, 95% CI: 0.283-0.964). This association was also slightly seen for PAR3 (Hazard Ratio: 0.695, 95% CI: 0.390-1.236) and PAR4 (Hazard Ratio: 0.602, 95% CI: 0.334-1.087) but without getting statistically significant. For PAR2 protein, we found rather the opposite effect with a trend to a worse survival when patients have a low intra-tumoral PAR2 protein expression (Hazard Ratio: 1.479, 95% CI: 0.827-2.644). Based on published different functional actions of PAR1 and PAR2 on proliferation of GBM cells (25) and the herein found different effects on survival time of GBM patients, we investigated the influence of a high PAR1 (> median) combined with a low PAR2 (< median) expression. This combination resulted in an even worse survival scenario (Hazard Ratio: 0.341, 95% CI: 0.142-0.820) than a solely high protein content of PAR1 (Hazard Ratio: 0.522, see above).

Given the found association of a high individual PAR1, PAR3 and PAR4 protein content with a worse patients´ prognosis and the knowledge of PAR heterodimers (26), we next asked if a combined consideration of these PAR subtypes increased the relative risk of a shortened survival time. For this analysis, patients were again divided in subgroups depending on the fact that two of the PAR subtypes were at high or low expression (< median versus ≥ median). As shown in **Supplementary Figure S3A**, a high protein content of both PAR1 and PAR3 (Hazard Ratio: 0.0383, 95% CI: 0.162-0.907), PAR1 and PAR4 (Hazard Ratio: 0.453, 95% CI: 0.213-0.962) as well as PAR3 and PAR4 (Hazard Ratio: 0.389, 95% CI: 0.158-0.916) was associated with a particular worse survival time. In contrast and as expected from the Kaplan Meier single protein analysis, for the combination of PAR2 with PAR1, PAR3 or PAR4, we did not observe any difference in the survival curves.

Since there seems to be a link between some of the PAR subtypes, we analyzed if there is a correlation between the protein expression of the individual PAR subtypes. Using again Spearman correlation analyses, we detected a positive correlation between PAR1 and PAR4 protein content (r^2^ 0.45, 95% CI: 0.197-0.65) matching the potentiated influence on survival time of GBM patients. In contrast, a slight negative correlation was found for PAR2 and PAR4 protein content (r^2^ -0.39, 95% CI: -0.609-0.130). The other PAR protein subtype combinations did not show any significant correlation (**Supplementary Figure S3B**).

### Differences in PAR mRNA and protein expression in stem-like GBM neurospheres

Since it is hypothesized that stem-like GBM cells are discussed to be responsible for therapy failure and recurrence in GBM patients, we firstly assessed the mRNA expression of the PAR subtypes in adherent LN-18 cells and LN-18 neurospheres, which are thought to be enriched in cancer stem cells. We compared expression of PAR mRNA over four passages of adherent (LN-18-adh) and neurosphere (LN-18-NS) culture with the parental cell state (LN-18-par) at the start of the experiment.

**Figure 3:**
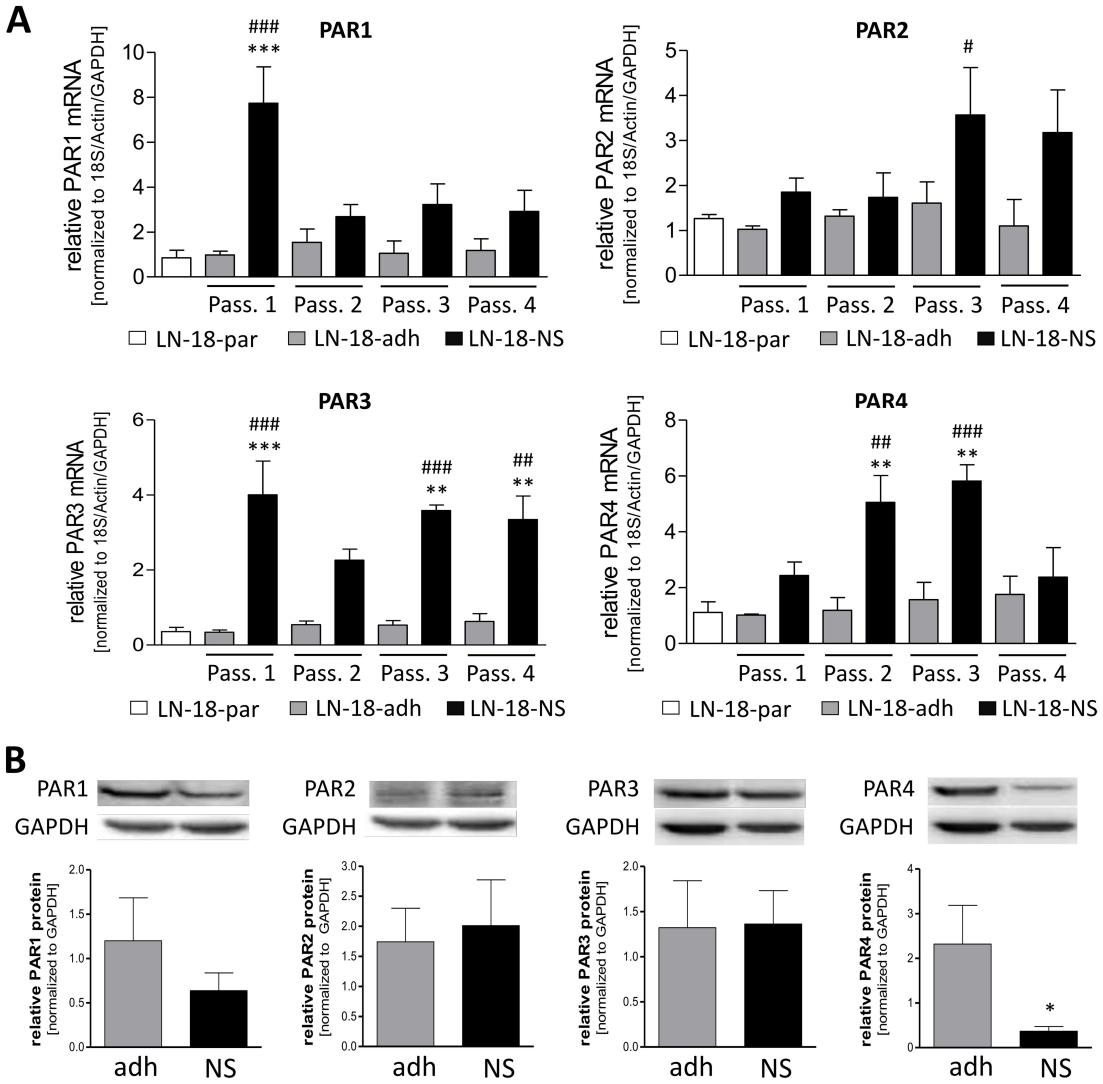
PAR1–4 mRNA and protein content in adherent and neurospheric LN-18 GBM cells. **(A)** Comparative PAR1–4 mRNA expression of both adherent (LN-18-adh, grey bars) and neurospheric (LN-18-NS, black bars) LN-18 cells of the passages one to four (Pass. 1-4) as well as of parental LN-18 cells (LN-18-par, white bars). Gene expression was measured by qPCR. Each mRNA level of the target genes (PAR1-4) was normalized to the mean of 18S rRNA, GAPDH and β-actin using the 2^-ΔΔct^ method, n=4. **(B)** Comparative PAR1–4 protein content of both adherent (LN-18-adh, grey bars) and neurospheric (LN-18-NS, black bars) LN-18 cells of passage three. The protein content of the respective PARs was determined by western blotting and normalized to the housekeeping protein GAPDH. Representative immunoblots are shown above the diagram, n=3-4. OneWay ANOVA with Bonferroni’s Multiple Comparison Test, *p < 0.05, **p < 0.01 and ***p < 0.001 LN-18-adh vs. LN-18-NS; #p < 0.05, ##p < 0.01 and ###p < 0.001 LN-18-adh vs. LN-18-NS.

As shown in **Figure 3A** by the presented qPCR data, all PAR subtypes were up-regulated in LN-18 neurospheres but to a different extent. The mRNA expression of PAR1 was highest in LN-18 neurospheres at the beginning of the experiment with an about 8-fold increase in passage 1 compared the parental and adherent counterpart. This up-regulation of PAR1 was much less in the passage 2 to 4. In contrast, PAR2 mRNA was not elevated in neurospheres of passage 1 and 2, but was increased about 3-fold compared to adherent LN-18 cells in passage 3. Based on the qPCR data of **Figure 3A**, PAR3 mRNA content was stably up-regulated (3- to 4-fold) in LN-18 neurospheres at all investigated passages. The mRNA expression results further showed that PAR4 is significantly increased about 5-fold in LN-18 neurospheres of passage 2 and 3 which was then normalized to the adherent counterpart at passage 4.

We next assessed the protein levels of PAR subtypes by immunoblotting technique in adherent and neurospheric LN18 cells at passage 3. As demonstrated in **Figure 3B**, all PAR subtypes could be detected on the protein level in both adherent and neurospheric LN-18 cells. Neither PAR1, PAR2 or PAR3 protein expression were significantly changed in LN-18 neurospheres compared to the adherent counterpart. Unlike, we found a significant decrease of PAR4 protein level in LN-18 neurospheres from 2.32 (± 0.87) to 0.37 (± 0.11) for what fits the patient data with a reduced PAR4 expression in GBM specimen.

### PAR inhibition results in a reduced viability of adherent GBM cells but not of stem-like neurospheres

**Figure 4:**
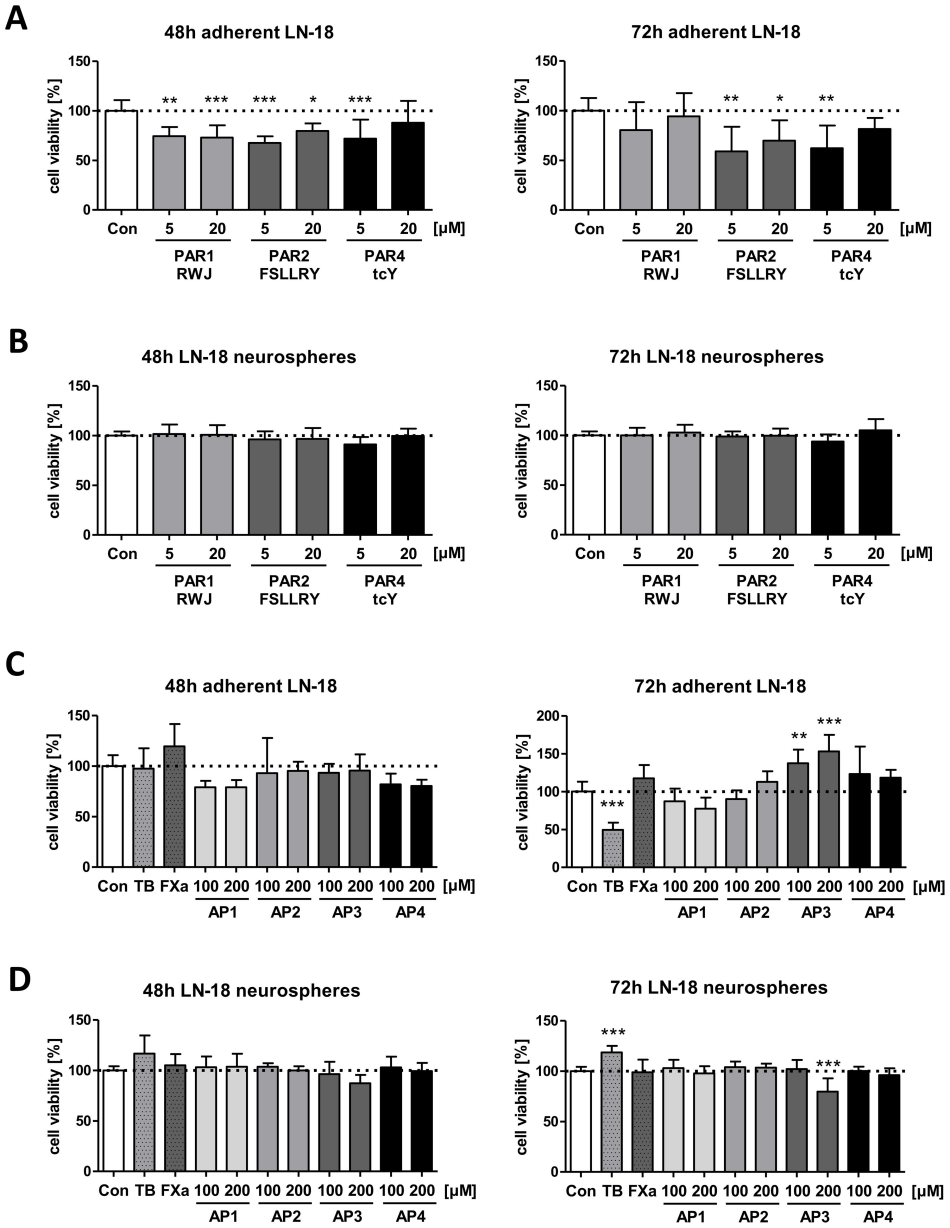
Impact of PAR subtype specific inhibitors and agonists on viability of LN-18 GBM cells. Adherent and neurospheric LN-18 cells of passage 1 or 3 were treated for 48 or 72h with the respective compounds followed by measurement of cell viability using the Resazurin assay. (A+B) Treatment of adherent **(A)** and neurospheric **(B)** LN-18 cells with inhibitors of PAR1 (RWJ), PAR2 (FSLLRY) and PAR4 (tcY) (each 5 and 20 µM), n=3-4. (C+D) Incubation of adherent **(C)** and neurospheric **(D)** LN-18 cells with Thrombin (TB, 30 U/ml), Factor Xa (FXa, 30 nM) or PAR subtype specific agonists (AP1 to AP4, each 100 and 200 µM), n=3-4. OneWay ANOVA Dunnett’s Multiple Comparison Test, *p < 0.05, **p < 0.01 and ***p < 0.001 vs. Con.

We next examined the effect of PAR inhibition on cell viability of both adherent and neurospheric LN-18 cells to evaluate whether these receptors might be structures for targeted therapeutic options in GBM treatment. Viability of adherent LN-18 cell was significantly reduced 48 and 72h after application of specific antagonists of PAR1 (RWJ), PAR2 (FSLLRY-NH2) and PAR4 (tcY-NH2) (**Figure 4A**). Since a specific PAR3 antagonist was not available, we could not analyze the impact of this PAR subtype on growth of GBM cells. In the presence of the selective PAR1 antagonist RWJ, the viability of adherent LN-18 cells was decreased to 74.5% (5 µM) and 72.9% (20 µM) after 48h, which was tendencially normalized to control cells maybe based on degradation of the compound. The selective PAR2 antagonist FSLLRY-NH2 caused a similar reduction in viability of adherent LN-18 cells to 67.7% (5 µM) and 79.7% (20 µM) after 48h as well as to 59.1% (5 µM) and 69.9% (20 µM) after 72h, respectively. The viability reducing effect of the selective PAR4 antagonist tcY-NH2 was only at 5 µM significant with values of 71.9% (48h) and 62.2% (72h). In contrast to the reduced viability of adherent LN18 cells upon PAR antagonists, stem-like LN18 neurospheres did not react to the application of either of the PAR antagonists (**Figure 4B**).

We further examined if treatment of LN18 GBM cells with PAR activating peptides (AP) specific for each subtype (AP1, AP2, AP3 and AP4) as well as of thrombin (PAR1, 3 and 4 agonist, 30 U/ml) and Factor Xa (PAR1 and 2 agonist, 30 nM) modulates viability of adherent or neurospheric LN18 cells (**Figures 4C, D**). Among the specific activating peptide (AP), only application of AP3 caused a change in the viability of LN-18 neurospheres to 79.7% after 72h whereas a significantly increased viability was seen for adherent LN-18 cells to 137.3% (100 µM) and 153% (200 µM). Interestingly, we also found contrasting results for treatment of adherent and neurospheric LN-18 cells with Thrombin (TB, 30 U/ml). 72h after application of thrombin, a significant decrease in viability of adherent LN-18 cells to 49.6% was observed. In contrast, in LN-18 neurospheres, a slight viability-promoting effect (118.6%) was present LN-18 neurospheres (**Figure 4D**).

## Discussion

Hypercoagulopathy is a common feature in patients with glioblastoma (GBM) associated with a significantly increased thrombin production (27, 28). Thrombin promotes spreading of tumor cells, stimulates tumor growth and angiogenesis - all processes that can contribute to the characteristic features of GBM (7). The cellular effects of thrombin are mediated via protease-activated receptors PAR1, 3 and 4, which promote proliferation and migration of cells and whose expression correlates directly with the invasiveness of tumors (29).

Based on this observation, we performed an extensive analysis of PAR1–4 mRNA and protein expression in human GBM samples from primary and recurrent tumors compared to non-malignant brain. To exclude the influence of previous GBM therapy on PAR expression, only primary GBM specimen were used for Kaplan Meier survival analyses.

Expression of PAR subtypes was neither associated with the age of the patients at diagnosis nor the sex of the patients. We found a significant upregulation of PAR1–3 mRNA in both primary GBM and relapsed tumor samples. This finding could not be confirmed at the protein level, there was no difference in PAR1–3 protein content between non-malignant brain and GBM. No significant correlation was seen between mRNA and protein content of either of the PARs. In contrast to PAR1-3, the PAR4 mRNA was unchanged but PAR4 was significantly downregulated at the protein level.

Overexpression of PAR1 mRNA in GBM demonstrated in our study is consistent with other studies, with PAR1 being one of the most intensively studied PAR subtype in GBM (16, 19, 30). PAR1 is also functionally expressed in healthy brain tissue (31) and PAR1 expression in astrocytoma increases with the degree of malignancy (32). We could not find any association of PAR1 mRNA with patients´ survival or PAR1 protein content. Interestingly, although PAR1 protein expression was not increased in GBM samples, a significant correlation with patient´s survival was observed. Patients having a high PAR1 protein level showed a significant shorter overall survival time which is in accordance with data from Zhang and colleagues who showed that PAR1 protein in malignant glioma is correlated with both the malignancy grade and a shortened survival of patients (17). Of note, the association with a shortened survival time found in our study is even more pronounced when patients have both a high PAR1 and PAR3 or PAR4 protein level (Hazard ratios: 0.522 vs. 0.383 or 0.453). Also, a combined high expression of PAR3 and PAR4 resulted in a worse overall survival (Hazard ratio: 0.379) arguing for a combined role of these receptor subtypes. Ultimately, these results show that the PAR subtypes PAR1, 3 and 4, but not PAR2, possibly influence each other through dimerization or have a synergistic effect in the disease progression by activating similar signaling pathways which mutually reinforce their pro-tumoral effects. In contrast, the positive effect of high PAR2 protein expression on patient survival may be cancelled out by interaction with the other PAR subtypes.

Regarding PAR2, Luo and colleagues showed overexpression of PAR2 mRNA in the GBM cell line U-87MG (21), while Carneiro-Lobo et al. could not find any differences between GBM and non-tumoral brain tissue (32). In the present work, only PAR2 mRNA but not PAR2 protein was elevated in GBM compared to NMB. It has been described that activation of PAR2 induces cell proliferation, angiogenesis and cell motility and contributes to tumor cell metastasis. Thus, a high PAR2 expression, as with PAR1, could have a pro-tumorigenic effect in GBM. In contrast, we found a trend toward prolonged overall survival in patients with high PAR2 protein levels (Hazard ratio: 1.479, p=0.18). Of note, for PAR1 and PAR2 different actions on GBM cells are shown with inhibition of proliferation by a specific PAR2 agonist while this was not seen by a PAR1 agonist. Stimulation of both PAR-1 and PAR-2 resulted in a similar [Ca2+]i response, while the effects on cell proliferation and activation of PKC isozymes were distinct, suggesting that these receptor subtypes activate different signal transduction pathways (25). In addition, PAR1 and PAR2 also have opposite effects on the proliferation and migration of PC3 prostate cancer cells (33). Our findings of improved survival with high PAR2 expression seem to be consistent with these data. Interestingly, these results are underlined by the even worse survival of GBM patients with high PAR1 and low PAR2 expression (hazard ratio: 0.341, p=0.016) compared to those with only high PAR1 protein (hazard ratio: 0.522, see above). This suggests that different signal transduction pathways may be activated by PAR1 and PAR2 in GBM cells.

However, Ostrowska et al. were able to detect a functionally active PAR3 on glioma cells (23). Other groups could not detect PAR3 and PAR4 in glioma cells (12, 24). Our study shows that PAR3 protein is expressed in GBM but without a significant change in comparison to non-malignant brain tissue. No influence on the survival time of patients with GBM could be demonstrated for PAR3 in our study either. Similar to PAR3, PAR4 has rarely been studied in GBM to date. Functional expression of PAR4 was shown in astrocytoma cells (34). Our study confirmed PAR4 protein expression in GBM, but PAR4 expression was significantly lower compared to non-malignant brain tissue suggesting that this PAR subtype might play a minor role in GBM tumor progression. Of note, patients with low PAR4 protein expression showed a slightly better prognosis but this was not statistically significant.

As mentioned above, when PARs were considered individually, only PAR1 protein showed a significant association with patient´s survival. Patients with high PAR1 protein had significantly shorter survival times (Hazard of 0.52) suggesting PAR1 as a main mediator of GBM tumor progression. Regarding PAR2 protein, a trend to a longer survival was seen when tumor samples of GBM patients bear a higher expression. This probably explains the fact that their respective effects on survival were cancelled out when PAR1 and PAR2 are considered together. Another interesting finding was that a combined low protein expression of PAR1 and PAR3, PAR1 and PAR4 as well as of PAR3 and PAR4 was associated with a prolonged survival time of GBM patients. In accordance with our data, PAR1 expression was found to be correlated with decreased survival in GBM patients by another group (17).

However, PAR1 was significantly increased in GBM samples at the mRNA level, but this was not transferred to the protein level. In contrast, PAR was found to be unchanged at the mRNA level but the PAR4 protein content was significantly reduced in GBM. Interestingly, for both PAR1 and PAR4, a high protein content was associated with a worse patient´s survival which was significant only for PAR1 protein whereas for PAR4 just a trend was seen. This may indicate a more important role of PAR1 in the progression of GBM. PARs can form both homo- and heterodimers with themselves or other PAR subtypes to regulate signaling and activation (35). PAR1 constitutes stable heterodimers with PAR4 which is required for efficient PAR4 cleavage by thrombin (36). So, an inefficient heterodimerization between both receptor subtypes or a preferred formation of PAR4-PAR4 homodimers may results in diminished PAR4 activation. In addition, PAR1 is a high-affinity receptor for thrombin whereas PAR4 is a low-affinity receptor, but partial functional redundancy between PAR1 and PAR4 is suggested (37). Thus, PAR1 could be the more relevant PAR subtype for stimulation of GBM cell proliferation, migration and therapy resistance especially as it also seems to have an importance for tumor-initiating progenitor cells (19) which was not described for PAR4. PAR1 might also have more potent impact on those pro-tumorigenic pathways as PAR4, e. g. in a similar way as shown for stronger effects of PAR1 vasculogenesis compared to PAR4 (38). However, PAR4 is less well studied in cancer but might act synergistically with PAR1 which could explain the poorer patient outcome when both PAR subtypes are elevated.

Of note, opposite roles of PAR1 and PAR4 are also described and it was speculated that PAR4 is low expressing because it is a very potent receptor, at least in endothelial cells (39). Further, in esophageal squamous cell carcinoma, PAR1 and PAR4 were shown to have opposite effects on tumor growth, with PAR1 promoting tumor growth and metastasis, while PAR4 has an inhibitory impact (40). However, an activation of both PAR1 and PAR4 may result in modulation of different pro-tumoral cascades and to varying degrees which may also dependent on the cell type.

Until now, a tumor-suppressive function of PAR4 is highly speculative as only one publication has demonstrated an inhibitory effect of PAR4 on tumor growth in esophageal squamous cell carcinoma (40). However, a reduced PAR4 protein expression in GBM specimen and stem-like GBM cells, as observed in our study, would be consistent with a tumor-suppressive role. But of note, a high PAR4 protein content was rather associated with a worse overall survival in our patient cohort. From this we cannot conclude that PAR4 acts as a tumor suppressor in GBM.

Furthermore, in contrast to PAR1, PAR4 expression dynamically adapts to various stimuli including thrombin, angiotensin II, sphingosine-1-phosphate, high glucose and redox stress, suggesting that PAR4 level is switched on ‘on demand’. PAR4 is discussed to be a kind of sensor of pathological stress which is associated with a strong up-regulation from normally low expression under resting conditions (41). Therefore, it can be speculated that the reduced PAR protein level found in GBM samples in our study is only a snapshot and might change depending on the conditions in the tumor microenvironment, particularly under stress condition such as hypoxia. This could also explain why PAR1 is high in GBM but PAR4 is low, and why patients in whom both receptor subtypes are highly expressed together have poorer survival than patients in whom only one of the two is highly expressed. However, our work is the first one that performed both a single and combined evaluation of all PAR subtypes in GBM patients.

It is assumed that a subpopulation of GBM cells with stem cell-like properties is responsible for tumor recurrence (42). PAR1 inhibition suppresses the self-renewal and growth of A2B5-defined glioma progenitor cells and their derived gliomas in a mouse model (19). Another group also demonstrated that PAR1 blocking inhibits proliferation and invasion of GBM cells *in vitro* and prolongs survival in an GBM animal model (43). LN-18 cells can be used as *in vitro* model for cultivation of both adherent and stem-like neurospheric cells (44, 45). To test whether there are variable expression levels over time during the cultivation of the stem-like LN-18 neurospheres, PAR mRNA expression was analyzed at different passages. Indeed, we found some differences in the time course of mRNA increase in stem-like LN-18 neurospheres. PAR1 mRNA was strongly induced at passage 1 and then declined. In contrast, PAR4 mRNA was elevated at the time when PAR1 was reduced again, while PAR3 mRNA was elevated at all passages and PAR2 mRNA only increased at later time points. These differences could be due to different transcriptional regulation or a significantly modulated mRNA half-life, as has already been shown by other groups (46–49). On the other hand, an initially high expression of PAR1 may indicate an involvement in the development of stem cell properties which is consistent with the fact that PAR1 appears to be relevant for tumor-initiating progenitor cells (19). An increase over time, as observed for PAR2 and PAR4, may indicate a role in the maintenance or proliferation of stem-like cells. However, the precise role of these PAR subtypes in stem cell behavior has not been clarified to date. Considering that the expression levels of all PARs were slightly elevated in LN-18 neurospheres compared to the adherent cells, this may also reflect their ability to promote cell-cell adherence more than cell-substrate adherence, which is important for collective cell migration. However, at the protein level, the neurospheric expression of PAR1, PAR2 and PAR3 was almost identical to that in adherent LN-18 cells, and only the PAR4 protein was significantly decreased, which is consistent with the reduced PAR4 protein levels in the GBM specimen.

Next, we investigated whether the targeted stimulation of PAR subtypes using thrombin, Factor Xa, and activating peptides (AP1-4), which mimic the natural tethered ligand and bind specifically the receptor’s ligand-binding site externally to activate the respective PAR subtype, has any influence on LN-18 cell viability (50). Whereas Factor Xa had no significant influence on LN-18 cell viability, thrombin showed differences on adherent and neurospheric cells. Involvement of thrombin in the pathogenesis of GBM was already described in detail in a review article by Krenzlin et al. in 2017 (7). Of note, in our study thrombin increased viability of stem-like LN-18 neurospheres while treatment of adherent LN-18 cells resulted in a strongly diminished cell viability. Apoptosis-inducing effects of thrombin have also been shown in neurons and astrocytes at concentrations of 40–100 U/ml (51, 52). We used a thrombin concentration (30 U/ml) which is in a similar range. Differential effects of thrombin on the rat glioma cell line C6 were reported with mitogenic effects at high concentrations and apoptosis induction at lower concentrations (53). Ahmad et al. also have shown that thrombin promotes proliferation at low concentrations, whereas at higher concentrations thrombin inhibits tumor cell proliferation (54).

Thrombin is a potent activator of integrins such as the β3-integrin glycoprotein IIb/IIIa (55). Integrin heterodimers, consisting of non-covalently associated α and β subunits, are highly expressed in glioma stem cells and are critically involved in their self-renewal, differentiation, and pronounced resistance to drugs and chemo-radiotherapy through mechanisms involving cell adhesion and signaling (56). Further, glioma stem cells (GSCs) can activate platelets by producing thrombin. This endogenous coagulation cascades of GSCs is tumorigenic and promotes stemness and proliferation *in vitro* and its pharmacological inhibition delays tumor growth *in vivo*, too (57). Based on all of the above data on thrombin, it is conceivable that thrombin promotes the survival and viability of stem-like LN-18 cells, while the apoptosis-inducing effects of thrombin are in the foreground in adherent LN-18 cells resulting in a reduced cell viability.

In contrast to thrombin which activates both PAR1, PAR3 and PAR4, respectively, the activating peptide AP3 specifically activates PAR3. Unfortunately, PAR3 has only been researched to a very limited extent in cancer and GBM. Application of AP3 increased the viability of only adherent LN-18 cells, but not of the stem-like counterpart, despite both cell types express this PAR subtype at a similar protein level. This might argue for distinct and cell type specific downstream signaling pathways activated by PAR3 leading to either promotion or inhibition of cell viability.

In contrast, the other activating peptides didn´t cause a modulation of LN-18 viability. But we cannot exclude that an extended treatment period might result in viability modulating effects.

In addition, we performed inhibition experiments with receptor antagonists for PAR1, PAR2 and PAR4 (50). No specific antagonist is yet known for PAR3, so it was not possible to investigate the effect of its inhibition on LN-18 cells. Interestingly, the antagonism of either PAR1, PAR2 or PAR4 caused a significant reduction in the viability of adherent LN-18 cells. This partially corresponds to already published data showing that inhibition of PAR1 reduces the growth of tumor cells (19, 30). Such proliferation-inhibitory effects in adherent GBM cells have not yet been published for blocking of PAR2 and PAR4. In contrast to the adherent tumor cells, LN-18 neurospheres did not respond to inhibition of either PAR1, PAR2 or PAR4. This underlines the special character of tumor stem-like cells, which are believed to be responsible for the recurrence of GBM due to their resistance to chemotherapy (42).

Taken together, our study suggests the potential therapeutic and prognostic significance of PARs in GBM. With regard to therapeutically applicable methods, inhibitors such as those used in this study play a crucial role and should continue to be an essential part of studies on the importance of PAR proteins as therapeutic targets. Nevertheless, the data collected in our study question the success of therapy with PAR antagonists alone, as it is possible that stem cell-like tumor cells survive and trigger a recurrence.

## Methods

### Patient specimens

Following an institutional review board–approved protocol (Ethics Committee at the University Medicine Greifswald, Institute of Pharmacology) which is in accordance with the ethical standards of the Helsinki Declaration of the World Medical Association, human GBM tissues were collected from patients with primary GBM (n=94) or relapsed GBM (n=48) who underwent surgical removal of GBM within their therapeutic regime (study period from 15.10.2007 to 30.04.2017). Informed consent to participate was obtained from all study participants. Both male and female patients were included in the study. In terms of gender, there were more men than women in our cohort (1.35 times). Patients of all ages were included in the study. Patients who did not consent to the use of their data were dropped out. Overall survival time was defined as the time span from the date of diagnosis to the date of death. Patients were allocated to groups according to their PAR subtype expression. Complete blinding of the patients was not possible, as conclusions were drawn from clinical data, so that pseudonymisation was used. The detailed clinical characteristics are shown in **Table 1**.

**Table 1:** Clinical characteristics of the GBM patient cohort.

Glioblastoma patient cohort		
Tumor entity	% (n)	
GBM WHO grade IV	100 (142)	
Primary GBM	66.2 (94)	
Relapsed GBM	33.8 (48)	

Primary vs. relapsed GBM	Patients with primary GBM	Patients with relapsed GBM
Age at diagnosis	[years]	[years]
Median	67	58
Min.-Max.	14 – 86	25 – 85
Age classes	% (n)	% (n)
< 50 years	9.57 (9)	20.83 (10)
50-<60 years	23.40 (22)	35.42 (17)
60-<70 years	26.60 (25)	22.92 (11)
70-<80 years	31.91 (30)	18.75 (9)
> 80 years	8.51 (8)	2.08 (1)
Sex	% (n)	% (n)
Male	57.45 (54)	66.67 (32)
Female	42.55 (40)	33.33 (16)

Survival of patients with primary GBM	[Months]	
Median	12.4	
Survival rate	% (n)	
1-YSR	37.23 (35)	
2-YSR	7.45 (7)	
5-YSR	1.06 (1)	
Vital status unknown	17.02 (16)	

1/2/5-YSR, 1/2/5-year survival rate.

Further, non-malignant brain tissues (frontal/temporal lobes) from the Institute of Pathology/Department of Neuropathology of the University Greifswald were analyzed. These non-tumoral brain specimens were obtained during routine autopsy. Tissue samples were cut and frozen at minus 80°C immediately after removal. Causes of death were pneumonia, heart failure, sepsis, or carcinoma of pancreas, respectively. There were no neurological disorders. In addition, protein and RNA samples of two non-malignant (one frontal and one temporal lobe) specimens were obtained from BioChain Institute Inc. (Newark, CA, USA).

### Cultivation of GBM adherent cells and neurospheres

The maintenance of human adherent LN-18 glioblastoma cells (ATCC, CRL-2610, RRID: CVCL_0392) was performed in DMEM medium supplemented with 10% FCS, 2 mM glutamine and 1% NEAA solution (100×) (all from PAN Laboratories) at 37°C, 95% humidity and 5% CO2. Neurospheres, which have been shown to be enriched in cancer stem cells, were cultured using the NeuroCult NS-A Proliferation Kit (STEMCELL™ Technologies) according to the manufacturers’ protocol. Cells were cultured in 6-well plates. For obtaining and maintenance of neurospheres, LN-18 cells were centrifuged at 1.000 rpm for 3 min at room temperature. The supernatant was removed and cells were washed two times with pre-warmed PBS. Afterwards, the LN-18 cell pellet was resuspended in NeuroCult NS-A Proliferation medium supplemented with 10 ng/ml bFGF, 20 ng/ml EGF and 2 µg/ml heparin as suggested by the manufacturer. After counting cells, the appropriate cell number (300.000 cells/2 ml) was seeded in 6-well plates. Already after 2 days, the first neurospheres could be detected, but cells were cultured for a total of seven days until passaging. Every third or fourth day 0.5 ml fresh medium was added. According to the manufacturers’ protocol, neurospheres should be passaged when the reach approximately 100 to 150 µm diameter, typically this was the case after seven days of cultivation. For passaging, the free swimming neurospheres were collected followed by centrifugation at 1.000 rpm for 3 min at room temperature. The supernatant was removed except a remaining rest of about 100 µl. This remaining cell suspension was then pipetted up and down for 50 to 100 times to mechanically dissociate all of the neurospheres. The separated neurospheric cells were counted and seeded again into 6-well plates as described above. This dissociation procedure was performed for four passages. The LN-18 glioblastoma cell model used here was well characterized in a previous study by our group (45). LN-18 cells show a very good generation of neurospheres enriched in several potential stem cell markers such as Nestin, CD133, CD44 or CD95 whereas the astrocytic differentiation marker GFAP is reduced.

### Quantitative real-time PCR analysis

Total RNA was isolated using PeqGold RNAPure (PeqLap) and reversely transcribed using the High Capacity cDNA Reverse Transcription Kit (Applied Biosystems by Life Technologies, Weiterstadt, Germany). The following Gene Expression Assays on Demand from Applied Biosystems were used for qPCR: F2R FAM-Hs00169258_m1, F2RL1 FAM-Hs00608346_m1, F2RL2 FAM-Hs00765740_m1, F2RL3 FAM-Hs01006385_g1. qPCR was performed in a 7900 HT Fast Real-Time PCR system from Applied Biosystems using TaqMan™ Gene Expression Master Mix. Each mRNA level of target genes was normalized to glycerin-aldehyde-3-phosphate dehydrogenase (GAPDH) and β-actin using the 2^-ΔΔct^ method. Samples in which the c_T_ values of the housekeeping genes deviated by more than +3.3 from the mean value of all samples were excluded from further analyses.

### Western blot

Protein extracts of patient’s glioblastoma and non-tumoral brain samples were prepared using the Qiagen TissueLyser II (RRID: SCR_018623). Nitrogen-cooled tissue tumor samples were shredded for 90 seconds at a frequency of 30 Hz. Immediately after shredding, the resulting tissue powder was dissolved in precooled lysis buffer (50 mM Tris–HCl pH 7.4, 100 mM NaCl, 0.1% Triton X-100, 5 mM EDTA containing protease/phosphatase inhibitors: 1 mM PMSF, 1 mM leupeptin, 1 mM aprotinin, and 250 μg/ml sodium vanadate) and incubated on ice for 45 minutes followed by a centrifugation step at 6,000 rpm to remove cell debris. GBM cells from cell culture experiments were scraped or trypsinized, transferred to a 1.5 ml tube and centrifuged at 10,000 rpm for 3 min. Afterwards, cell pellets were resuspended in the same lysis buffer used for tissue specimens (see above). The BCA Protein Assay Kit (Thermo Fisher Scientific) was used to determine the protein concentrations in the lysates. Subsequently, after denaturation in Laemmli buffer at 95°C for 5 minutes, 40 μg of each sample was separated on 10% SDS polyacrylamide gels. For immunoblotting of the separated proteins to a nitrocellulose membrane (Whatman GmbH), the BioRad Trans-Blot SD Semi-Dry Transfer Cell (RRID: SCR_019036) was used. Afterwards, membranes were blocked with 5% skimmed milk/1% BSA in Tris-buffered saline containing 0.05% Tween 20 (TBST) for 1 hour at room temperature under shaking. Dilution (1:500) of the following primary antibodies was done in TBST and 0.05% sodium azide: rabbit anti-PAR1 (Santa Cruz Biotechnology Cat# sc-5605, RRID: AB_2101169), mouse anti-PAR2 (Santa Cruz Biotechnology Cat# sc-13504, RRID: AB_628101), mouse anti-PAR3 (Santa Cruz Biotechnology Cat# sc-393127, RRID: AB_3662749), mouse anti-PAR4 (Sigma-Aldrich Cat# SAB4503527, RRID: AB_10746877), and mouse anti-GAPDH (Meridian Life Science Cat# H86504M, RRID: AB_15154). Incubation with primary antibody was performed overnight at 4°C. Afterwards, the membrane was rinsed three times with TBST for five minutes each. The secondary horseradish peroxidase-conjugated goat-anti-mouse (Bio-Rad Cat# 1706516, RRID: AB_2921252) or goat-anti-rabbit IgG (Bio-Rad Cat# 170-6515, RRID: AB_11125142) antibodies were used at a 1:1000 dilution for 1 to 1.5 hours at room temperature followed by three times washing with TBST. Chemiluminescence signals were detected with Bio-Rad Chemidoc XRS Gel Imaging System (RRID: SCR_019690) using ECL Plus Western Blotting Substrate (Thermo Fisher Scientific) followed by densitometric analysis (Quantity One 1-D Analysis Software, RRID: SCR_014280). Each target protein was analyzed on a separate blot. Optical densities of the specific bands were calculated and normalized to GAPDH as a loading control.

### Cell viability analysis

GBM cells were seeded in 96-well plates with 10,000 cells per well. For experiments with adherent LN-18 cells, the medium was removed 24h later followed by incubation with the test compound for 48 or 72h. To establish the neurosphere culture, LN-18 cells were seeded at the same cell count, but the slowly growing neurospheres were cultivated for 96h and then treated with the respective test compounds. As specific PAR agonists we used the following PAR activating peptides: PAR1 activating peptide AP1 (TFLLR-NH2), PAR2 activating peptide AP2 (SLIGKV-NH2), PAR3 activating peptide AP3 (1-6) amide trifluoroacetate salt (TFAGAP-NH2), PAR4 activating peptide AP4 (AYPGKF-NH2) (all from Bachem AG). As specific PAR antagonists the following compounds were used: PAR1 antagonist RWJ, PAR2 antagonist FSLLRY-NH2, and PAR4 antagonist tcY-NH2 (all from Tocris Bioscience). Furthermore, we used Factor Xa (human plasma) as activator of PAR1, PAR2 and PAR4, as well as Thrombin as activator of PAR1, PAR3 and PAR4 (both from Enzo Life Sciences GmbH Lörrach, Deutschland).

After the respective incubation period, the medium was removed from adherent LN-18 cells, fresh medium containing 10% Resazurine (Fluorometric Cell Viability Kit I, PromoCell) was added, and cells were incubated for 1h at 37°C. Since neurospheres are floating structures, the neurosphere cultures have to be centrifuged for 10 min at 1,000 × g before the medium could be removed and the resazurin containing medium was added. As neurospheres show a lower metabolic activity, the incubation period was extended to three hours to obtain evaluable values. Fluorescence readings were recorded using a multiplate reader (Tecan Infinite M200, RRID: SCR_024560; excitation wavelength 530 nm, emission wavelength 590 nm). Data were calculated as percentage of cell viability of solvent treated cells.

### Statistical analyses

Statistical analyses were performed with GraphPad Prism 5.0 (GraphPad Software, RRID: SCR_002798). Data of *in vitro* analyses represent 3 or 4 independent experiments (indicated in the figure legends and shown as mean ± SD). Patients samples, showing gene or protein expression data, are shown as scatter plots with the Median. Pairwise comparisons were performed using Mann–Whitney U test. More than two groups were compared by OneWay ANOVA and Dunnett’s multiple comparison test or Bonferroni post test. Patient’s overall survival (OS) was defined as the time from the first tumor detection until death. Vital status and date of death were obtained from official population registry. GBM specimens were divided into the lower half versus the upper half of gene or protein expression level as determined by qPCR or immunoblotting technique (<Median vs. ≥Median expression). This subdivision was used as a basis for calculation of Hazard Ratios (<Median vs. ≥Median expression) and creation of Kaplan–Meier graphs which were compared by log-rank (Mantel-Cox) test. Statistical significances were defined as *p < 0.05, **p < 0.01, and ***p < 0.001.

## Data Availability

All data generated or analysed during this study are included in this published article (and its supplementary information files). All data is located in controlled access data storage at the University Medicine Greifswald. The raw data supporting the conclusions of this article will be made available by the authors, without undue reservation.
