# Supplementary material for: The protease-activated receptors are expressed in glioblastoma and differentially modulate adherent versus stem-like growth of LN-18 GBM cells
Source: Front Oncol. 2025 Jul 22;15:1582996. doi: 10.3389/fonc.2025.1582996 (PMC12321897; doi:10.3389/fonc.2025.1582996)
Supplement: Supplementary file 1 [file DataSheet1.pdf]

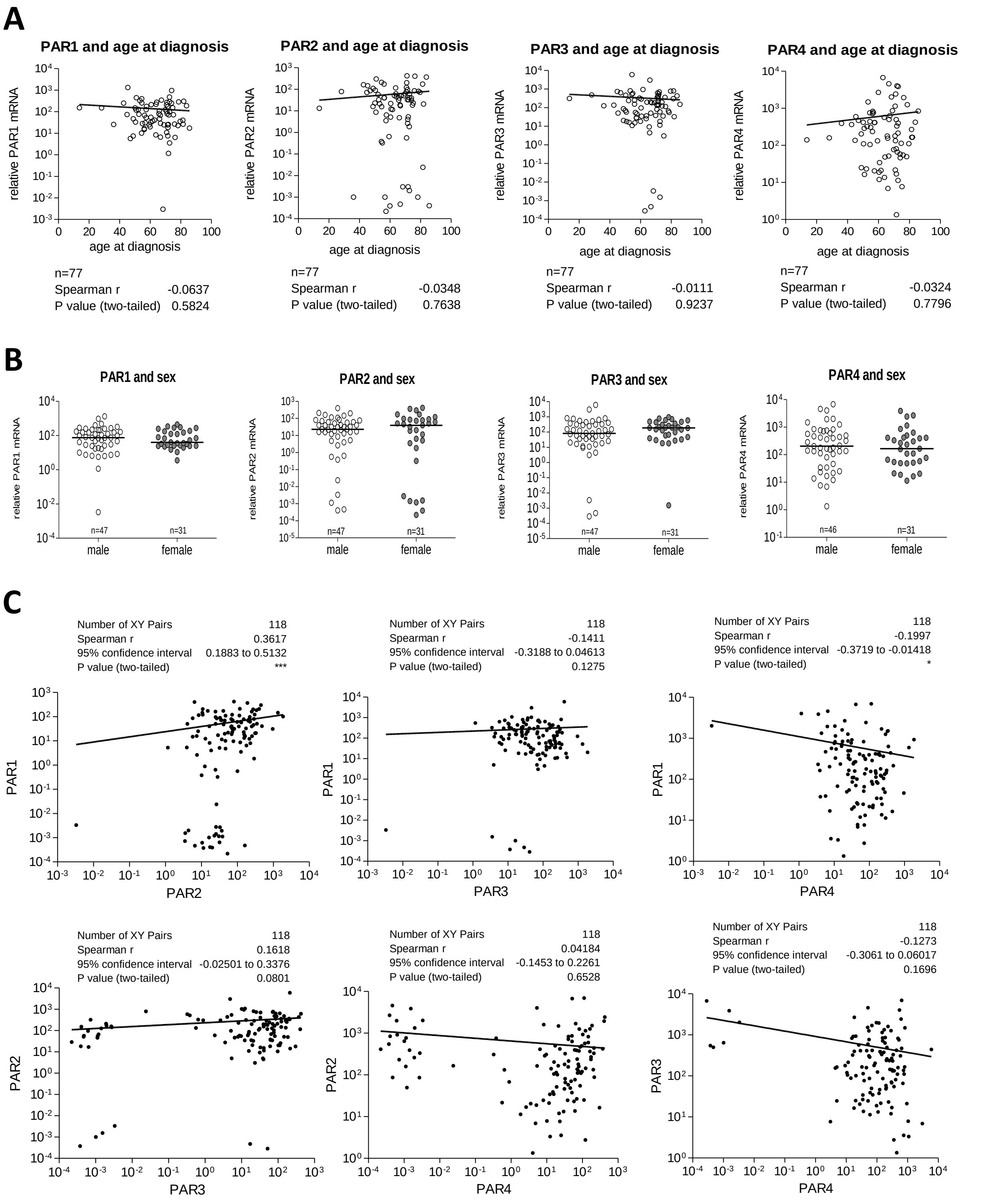

Supplementary Figure S1. PAR1-4 mRNA expression is not associated with patients age at diagnosis or sex. (A) Spearman correlation analyses (with non-linear regression curve) of patients age at diagnosis and PAR subtype mRNA expression based on qPCR data from primary GBM samples. (B) Primary GBM specimen were divided in male and female samples showing no

significant sex-dependent differences in mRNA expression of PAR1-4 based on qPCR data. Data are shown as scatter plots representing the median as horizontal bars, Mann Whitney U test. (C) Spearman correlation analyses (with non-linear regression curve) of mRNA expression data measured by qPCR to determine a potential association between PAR1, PAR2, PAR3 and PAR4 gene expression. *$p < 0.05$ and ***$p < 0.001$.

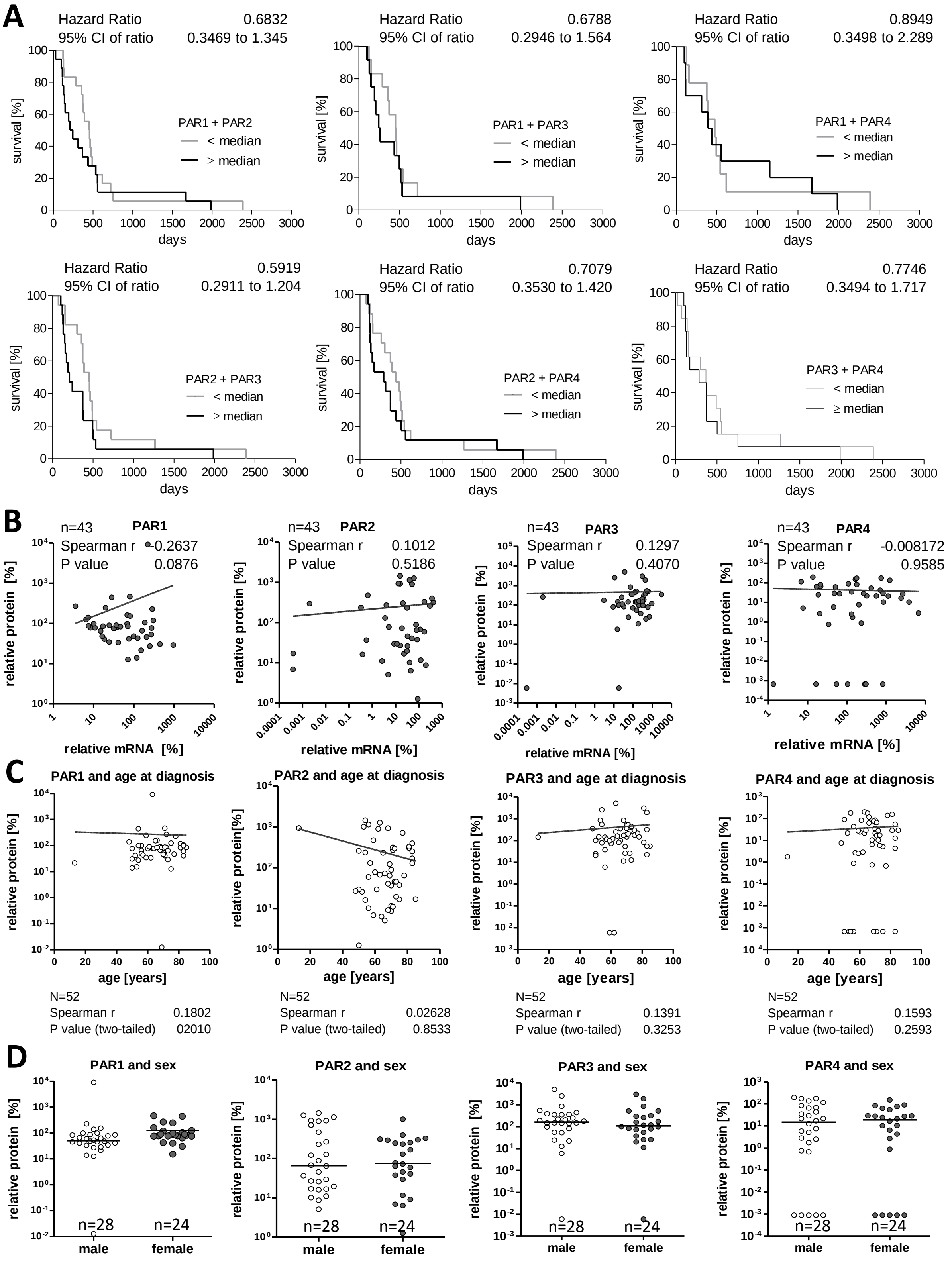

Supplementary Figure S2. (A) Kaplan Meier survival curves according to the combined mRNA expression of PAR receptors after subdividing the patients into quartile groups (high/high, low/low, high/low, and low/high). Only the combined groups high/high and low/low are shown. The gene

expression of the respective PARs was determined by quantitative RT-PCR and normalized to the combined expression of the housekeeping genes ß-actin and GAPDH. No significant associations were found. (B) Spearman correlation analyses between PAR1-4 mRNA (quantitative RT-PCR data) and protein level (western blot data). No significant correlation was found. (C) Spearman correlation analyses between PAR1-4 protein expression and the age at diagnosis. No significant correlation was found. (D) PAR1-4 protein level in GBM tissue after subdividing into male and female. No sex dependent differences in PAR1-4 protein content were found.

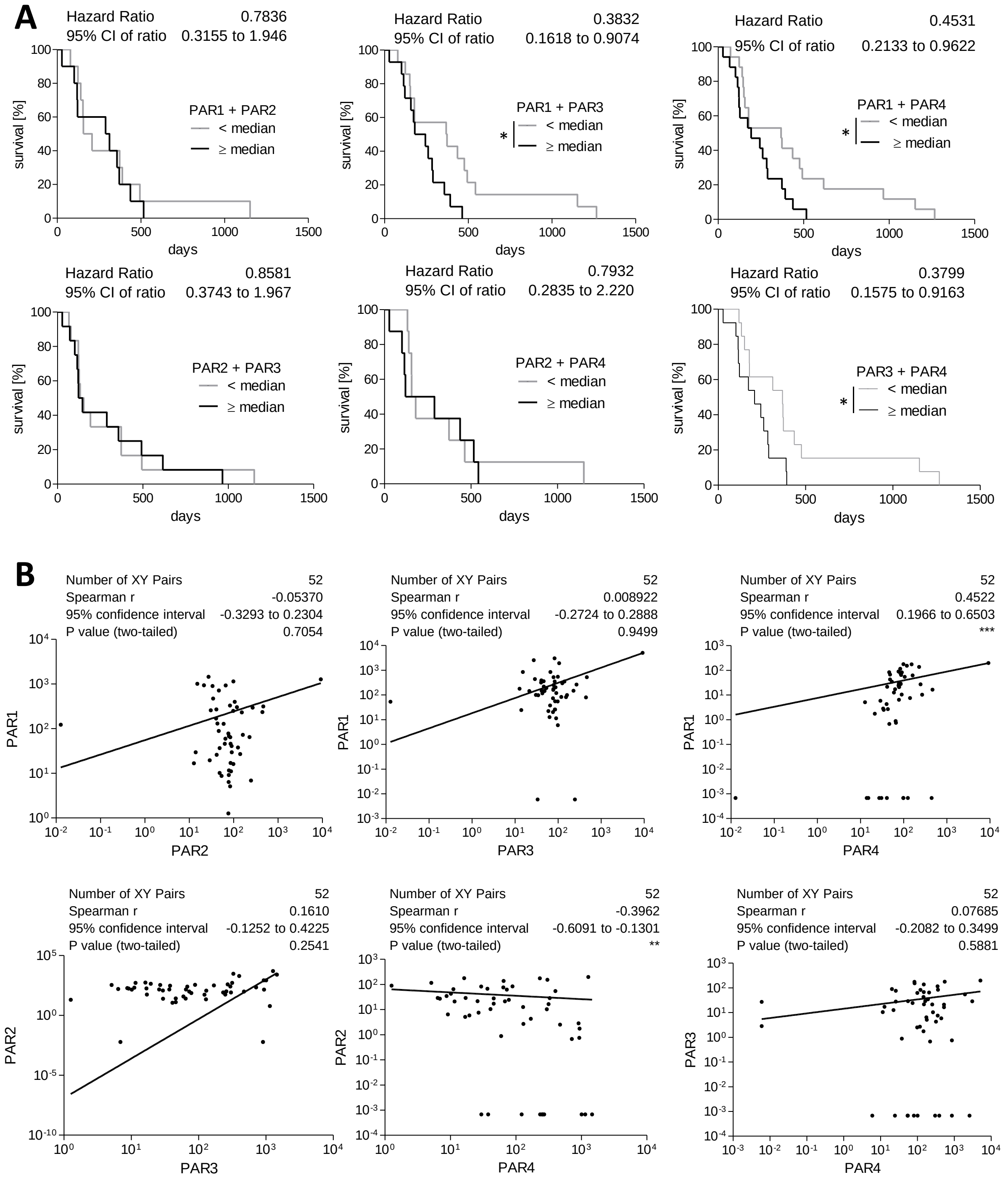

Supplementary Figure S3. (A) Kaplan Meier survival curves according to the combined protein level of PAR receptors after subdividing the patients into quartile groups (high/high, low/low, high/low, and low/high). Only the combined groups high/high and low/low are shown. The protein content of the respective PARs was determined by western blotting and normalized to the housekeeping protein GAPDH. Log-rank (Mantel-Cox) Test, *p < 0.05. (B) Spearman correlation analyses (with non-linear regression curve) of protein expression data measured by western blotting to determine a potential association between PAR1, PAR2, PAR3 and PAR4 protein content in GBM tissue. **p < 0.01 and ***p < 0.001.
